# Supplementary material for: Changes in cross-frequency coupling following closed-loop auditory stimulation in non-rapid eye movement sleep
Source: Sci Rep. 2020 Jun 30;10:10628. doi: 10.1038/s41598-020-67392-w (PMC7326971; doi:10.1038/s41598-020-67392-w)
Supplement: Supplementary file 1 — Supplementary information [file 41598_2020_67392_MOESM1_ESM.pdf]

Supplementary Information for

Changes in cross-frequency coupling following closed-loop auditory stimulation in non-rapid eye movement sleep

Abbreviated title: Auditory stimulation and cross-frequency coupling

Krugliakova Elena [a,b]*****, **Volk Carina** [a,b,c], **Jaramillo Valeria** [a,b], **Sousouri Georgia** [a,b], **Huber Reto** [a,b,d]

[a] Children's Research Center, University Children's Hospital Zurich, Steinwiesstrasse 75, 8032, Zurich, Switzerland

[b] Child Development Center, University Children's Hospital Zurich, Steinwiesstrasse 75, 8032, Zurich, Switzerland

[c] Center for MR-Research, University Children's Hospital Zurich, Steinwiesstrasse 75, 8032, Zurich, Switzerland

[d] Department of Child and Adolescent Psychiatry and Psychotherapy, Psychiatric Hospital, University of Zurich, Lenggstrasse 31, 8032, Zurich, Switzerland

*Corresponding author:
Elena.Krugliakova@kispi.uzh.ch

Supplementary Table 1. Sleep architecture.

Sleep parameters were calculated from the visual scoring of sleep stages. Sleep efficiency was calculated by dividing total sleep time with total time in bed. Wake after sleep onset is expressed as the percentage of total time in bed. Sleep stages (NREM sleep, stage N1, stage N2, stage N3, and REM sleep) are expressed as a percentage of total sleep time. P-values result from paired Student's t-tests. Bold letters indicate a significant difference ($p < 0.05$). Sleep architecture did not differ between the two nights except for total time in bed.

	**STIM**		**SHAM**		
	mean	SD	mean	SD	**p-value**
**Sleep architecture**					
Total time in bed [min]	451	21	468	12	**0.03**
Total sleep time [min]	410	35	430	22	0.08
Sleep efficiency [%]	91	5	92	3	0.63
Sleep latency [min]	24	19	18	9	0.31
Wake after sleep onset [%]	5	4	5	3	0.79
NREM sleep [%]	79	5	78	5	0.96
Stage N1 [%]	5	2	5	3	0.81
Stage N2 [%]	51	7	51	9	0.99
Stage N3 [%]	23	8	23	7	0.75
REM sleep [%]	21	5	21	6	0.96

Supplementary Table 2. Descriptive statistics about the number of stimuli presented and trials included in the analysis.

	STIM		SHAM		p-value
	mean	SD	mean	SD	
Total number of stimuli presented	1043	143	-	-	-
Stimuli presented during N1 [%]	0.03	0.07	-	-	-
Stimuli presented during N2 [%]	26.44	15.54	-	-	-
Stimuli presented during N3 [%]	73.10	15.63	-	-	-
Stimuli presented during REM [%]	0.02	0.08	-	-	-
Stimuli presented during wake [%]	0.39	0.40	-	-	-
Preprocessing step 1: Total number of N2 and N3 trials before manual artefact rejection	1039	145	1165	311	0.11
Preprocessing step 2: Total number of N2 and N3 trials after manual artefact rejection	1017	142	1153	310	0.09
Preprocessing step 3: Total number of N2 and N3 trials where stimulus was presented during rising part of the positive half-wave	638	111	666	186	0.53
Final proportion of N3 trials [%] (N2+N3=100%)	74	15	74	12	0.98

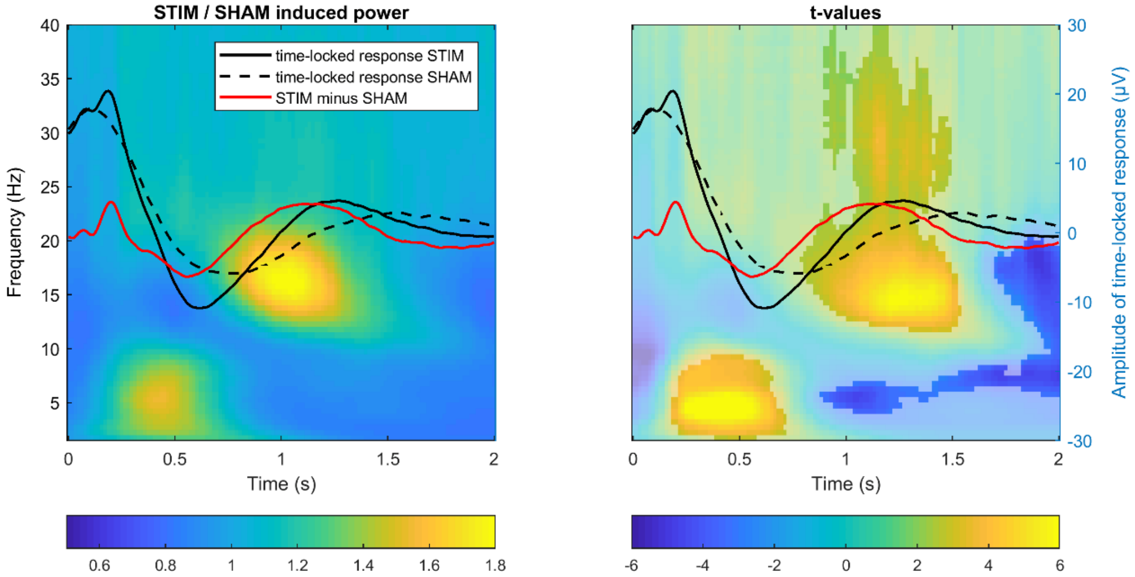

FIGURE S1. Induced power changes following the stimulus presentation during auditory closed-loop stimulation.

Superimposed grand-averaged ERP waveforms (in C4 target channel) for STIM (solid line) and SHAM (dashed line) conditions overlaid with (*left*) time-frequency power plot (relative change in power between STIM and SHAM collapsed across all channels) and (*right*) time-frequency t-values plot (shading indicates extent of significance, cluster corrected two-sided $p<.05$). Note that the induced power increase in theta and sigma bands is similar to the total power increase in Fig 2.

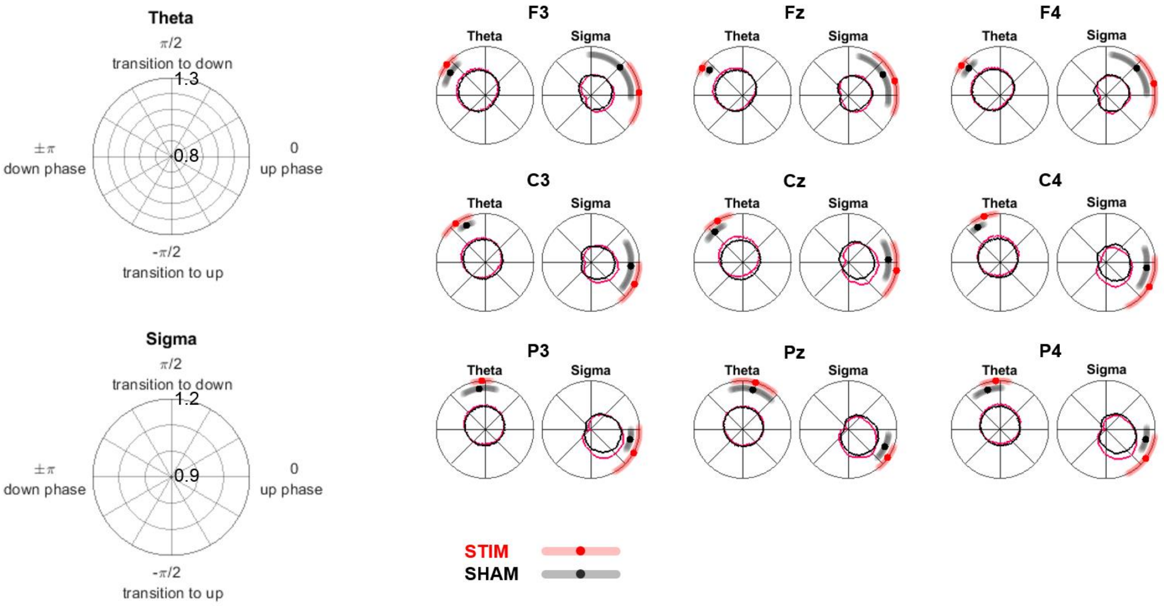

FIGURE S2. Delta-sigma coupling phases across 9 representative channels.

Dots and shading on polar plots display the mean preferred phase of delta waves (±STD) for 9 subjects. The radius of the circle inside the polar plot illustrates the dependence of the mean normalized amplitude of fast oscillation (theta or sigma) on the delta phase.

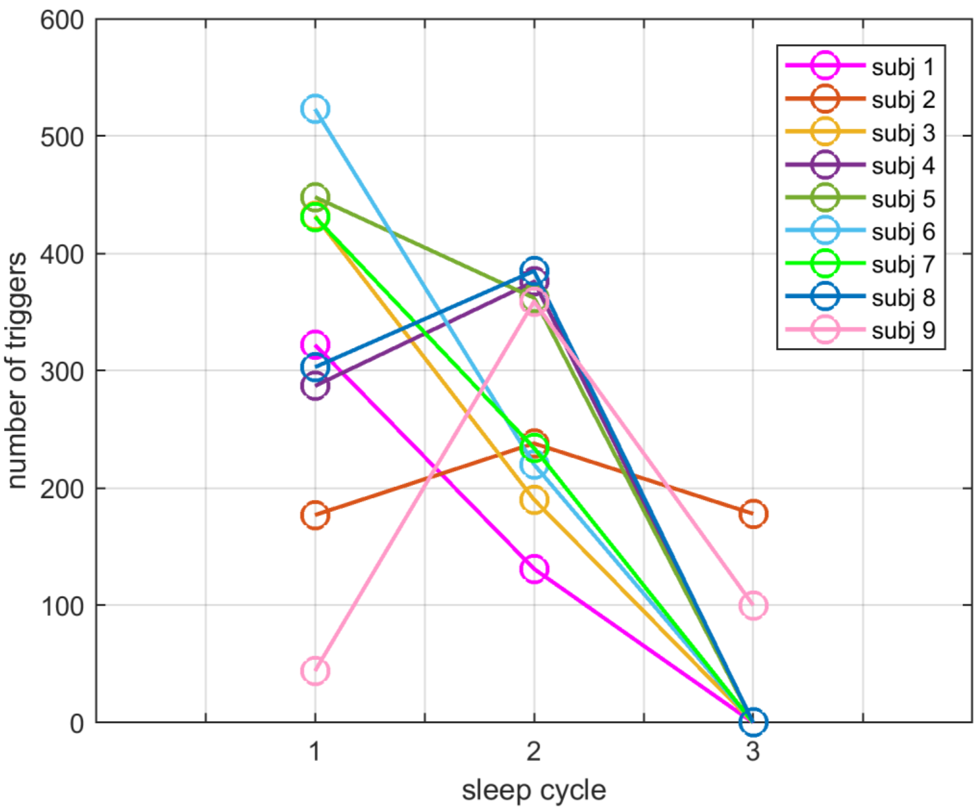

FIGURE S3. Distribution of stimuli across the first 3 sleep cycles in the STIM condition.

Individuals are color coded.
